# Peri- and Postoperative Treatment with the Interleukin-1 Receptor Antagonist Anakinra Is Safe in Patients Undergoing Renal Transplantation: Case Series and Review of the Literature

**DOI:** 10.3389/fphar.2017.00342

**Published:** 2017-05-31

**Authors:** Catharina M. Mulders-Manders, Marije C. Baas, Femke M. Molenaar, Anna Simon

**Affiliations:** ^1^Department of Internal Medicine and Expertise Center for Immunodeficiency and Autoinflammation, Radboud University Medical CenterNijmegen, Netherlands; ^2^Department of Nephrology, Radboud University Medical CenterNijmegen, Netherlands; ^3^Department of Nephrology, University Medical CenterUtrecht, Netherlands

**Keywords:** anakinra, interleukin 1, organ transplantation, renal transplantation, immunosuppressive drugs

## Abstract

In patients undergoing solid organ transplantation, the presence of an interleukin-1 (IL-1) driven disease may require the addition of IL-1 inhibiting drugs to the standard immunosuppressive regimen to protect against inflammation and negative graft outcome. Three patients undergoing renal transplantation were treated perioperatively with the interleukin-1 receptor antagonist anakinra. Kidney function increased rapidly in all three and the only complications seen were minor infections. *In vitro* studies report associations between serum and urinary levels of IL-1β and IL-1 receptor antagonist and negative graft outcome, and studies in animals and two small human trials illustrate a possible protective effect of anti-IL-1 therapy after solid organ transplantation. Peri- and postoperative use of anakinra is safe and effective in patients undergoing renal transplantation.

## Introduction

Patients undergoing solid organ transplantation depend on life-long immunosuppressive therapy with T-cel inhibiting drugs to prevent allograft rejection. In patients suffering from an interleukin-1 (IL-1) driven inflammatory disease, continuation of IL-1 inhibition is important because inflammation in these diseases is not controlled by the standard immunosuppressive drugs used after renal transplantation, and when anti-IL-1 directed therapy is stopped these patients are at high risk of recurrence of inflammation, associated with severe symptoms, such as arthritis, peritonitis, meningitis and many others.

IL-1 is a potent proinflammatory cytokine, which actions are mitigated by the circulating IL-1 receptor antagonist (IL-1RA). The recombinant IL-1RA anakinra is a very effective treatment for IL-1 driven diseases, such as autoinflammatory diseases and rheumatoid arthritis.

Little is known on the effects of perioperative IL-1 inhibition during renal transplantation. Hypothetically, the additional immunosuppressive effect of anti-IL-1 treatment on top of the standard immunosuppressive regimen could increase the risk of complications, especially infections. On the other hand, cessation of anti-IL-1 therapy in IL-1 driven diseases may lead to recurrence of systemic inflammation, which may negatively influence short- and long-term transplant function and in some diseases may increases the risk of amyloidosis in the allograft.

We have recently performed renal transplantation in three patients with IL-1 driven diseases while continuing anti-IL-1 therapy with anakinra before, during and after the transplantation^1^.

## Case series

### Case 1

A now 70-year old man developed recurrent fever episodes 12 years ago. One year after the first fever attack he developed end-stage renal disease. Renal biopsy at that time showed thrombotic microangiopathy (TMA). He was temporarily treated with plasmapheresis and was started on hemodialysis, which was later switched to peritoneal dialysis. After multiple episodes of fever and polyarthritis he was diagnosed with adult onset Still's disease (AOSD) and treatment with anakinra 100 mg on alternating days was started, which was later increased to the standard dose of 100 mg daily when renal function spontaneously increased. Four years after anakinra was started, this patient received a renal transplantation from a living related donor. He was started on immunosuppressive therapy with mycophenolate mofetil (MMF), tacrolimus and prednisone. Anakinra 100 mg daily was continued without interruption, only a single dose was skipped at the day of transplantation. After transplantation, kidney function improved rapidly and he could be discharged from the hospital 7 days postoperative. In the first year after the transplantation he experienced only two minor infections: an abscess located at an old drain entry site was drained 2 months after the transplantation and he was admitted for 5 days 6 months after the transplantation because of an upper respiratory tract infection. During these episodes anakinra and other immunosuppressive drugs were continued. 14 months after the transplantation this patient has been admitted for 34 days, and again 7 months later for 11 days, including several days in the intensive care unit, because of fever, encephalopathy and renal failure. These episodes were caused by exacerbations of the underlying AOSD. Two consecutive kidney biopsies showed endo- and extracapillary glomerulonephritis without signs of rejection or TMA. Treatment with high-dose steroids led to recovery of kidney function. Because of these severe exacerbations and apparent anakinra failure, anakinra was switched to the anti-interleukin-6 receptor antibody tocilizumab. The transplantation is now 2.5 years ago and this patient is doing well.

### Case 2

A now 22-year old woman was diagnosed with mutation negative cryopyrin-associated periodic syndrome (CAPS)/chronic infantile neurologic, cutaneous, articular (CINCA) syndrome at the age of 10. She had been suffering from rash, chronic meningitis, bone dysplasia and growth retardation since birth.

From the first year of life recurrent episodes of pyelonephritis had been present, associated with vesicourethral reflux, leading to impaired renal function and proteinuria. These had been progressive since the age of 17.

Directly after she was diagnosed with CAPS, she was started on anakinra. 8 years later she switched to the selective IL-1β antibody canakinumab. Two years later she developed end stage renal disease due to a combination of recurrent pyelonephritis, use of NSAIDs and hypertension. Kidney biopsy was contraindicated because of small kidney size, but there were no other signs of AA amyloidosis. A pre-emptive renal transplantation was planned and 2 months before, canakinumab was switched back to anakinra 100 mg on alternating days; the shorter half life of the latter makes it more easy to stop in case of complications. This patient received a renal transplantation of a living related donor almost 2 years ago. She was started on immunosuppressive therapy with MMF, tacrolimus and prednisone, while anakinra was continued. Kidney function increased rapidly and she could be discharged 6 days after the transplantation. Because of incomplete control of inflammation 4 months after the transplantation the dose of anakinra was increased stepwise to 100 mg each 36 h and later to 100 mg daily.

She is now doing well. She has been admitted three times since the transplantation: 3 months post-transplantation because of influenza and primo-Epstein Barr virus (EBV) infection (2 days) and both 8 and 11 months after the transplantation because of diarrhea, with positive norovirus PCR during the last episode. This may be related to her job at a children's day care center. MMF was switched to azathioprine because of diarrhea. She still uses anakinra 100 mg each day.

### Case 3

A 29-year old Turkish man was known with familial Mediterranean fever (FMF) since the age of 6 and slowly progressive nephrotic syndrome due to AA amyloidosis, which had been proven in a rectal biopsy at the age of 17. He had been on colchicine treatment since diagnosis, but without complete compliance and without proper medical guidance. He was only referred to a tertiary treatment center 11 years after the diagnosis of amyloidosis because of further deterioration of renal function after a pneumonia associated with vomiting and diarrhea with persistent use of ACE-inhibitors and NSAIDs. Hemodialysis had been initiated 1 month before referral. Renal biopsy at the time of referral showed AA amyloidosis. Because of unsatisfactory inflammatory control on colchicine, anakinra 100 mg on alternating days was started. This resulted in rapid control of inflammation. 15 months later he received a renal transplant from a living related donor. While continuing anakinra, he was started on standard immunosuppressive therapy with MMF, tacrolimus and prednisone. Renal function increased rapidly, but 4 days after the transplantation he developed a typical FMF-attack with fever, headache and malaise. The dose of anakinra was increased to 100 mg daily, because of suspected decreased plasma levels of anakinra due to increased renal clearance. This resulted in prompt resolution of symptoms. He was discharged 9 days after the transplantation. He is now 10 months after the transplantation. He has been admitted only once for 2 days 3 months after the transplantation because of pneumonia.

Six months after the transplantation, MMF has been switched to azathioprine on patient's request to simplify the drug regimen. He is doing well, has normal kidney function and still uses anakinra 100 mg daily.

## Discussion and literature review

### IL-1 inhibition during and after organ transplantation in patients with IL-1 driven inflammatory diseases

In three patients who underwent renal transplantation, the use of the IL-1RA anakinra in combination with MMF/azathioprine, tacrolimus and prednisone was safe and well tolerated. Few complications were seen; all were minor infections, which are common in renal transplant patients. Kidney function increased rapidly in all three patients and no drug-drug interactions were observed.

The only other patients undergoing renal transplantation with perioperative anakinra treatment reported in literature are two FMF patients described in a case report (Moser et al., 2009) and a case series (Ozcakar et al., 2016). Both had end stage renal failure due to AA amyloidosis. No complications were observed in either patient. Ozcakar et al. continued anakinra perioperatively, while it is unclear from the report of Moser et al. how they handled the drug in the perioperative period. In our three cases, we only skipped a single dose of anakinra on the day of transplantation. These scarce cases on continuing anakinra perioperatively in renal transplantation patients give an indication that it can be safely combined with immunosuppressants used to prevent allograft rejection.

There are several reports on the safety of anakinra when initiated after renal transplantation and other solid organ transplantations in patients with IL-1 driven diseases. A number of case reports and two case series describe 5 adults and adolescents with FMF and one adult with CAPS that were started on anakinra in combination with azathioprine or tacrolimus and prednisone 1–3 years after renal transplantation. All report no complications and good graft function (Leslie et al., 2006; Alpay et al., 2012; Celebi et al., 2014; Ozcakar et al., 2016). A seventh renal transplant patient treated with tacrolimus developed neutropenia 2 months after anakinra was started because of gout. The start of anakinra was also associated with decreased allograft function. It could be debated whether this really can be attributed to anakinra as this patient was 20 years post-transplantation and already known with chronic graft dysfunction (Direz et al., 2012).

All our patients received an allograft from a living related donor. Of the other reported patients that underwent renal transplantation during or after the initiation of anakinra, donor type was reported in only two: one patient that started anakinra after renal transplantation received a kidney from a living related donor (Leslie et al., 2006), while the other received an allograft from a deceased donor 5 months after the initiation of anakinra (Moser et al., 2009). In this latter patient, the immunsuppressive drug regimen post-transplantation was the same as in the cases described here (tacrolimus, MMF and prednisone) and there was immediate graft function (Moser et al., 2009).

Anakinra was also reported to be safe after liver transplantation and hematopoietic stem cell transplantation in one patient each (Petropoulou et al., 2010; Yilmaz et al., 2014). In all patients anakinra effectively reduced inflammation, reflected by rapid resolution of symptoms and decrease of serum inflammatory markers.

Our third case and a patient reported by Moser et al. (2009) illustrate that, equal to the need for dose adjustment in patients with impaired renal function, it is important to increase the dose of anakinra when renal function increases after renal transplantation to avoid recurrence of inflammation. Increased renal clearance of anakinra results in decreased anti-inflammatory effect. When decreased renal function was present, the patients reported here were treated with anakinra 100 mg every 36–48 h, depending on the severity of renal function loss, instead of the standard dose of 100 mg daily.

### IL-1 inhibition after organ transplantation in humans or animals in the absence of an inflammatory disease

In pancreatic islet transplantation, treatment with IL-1RA in apes (Danobeitia et al., 2015) or mice (Sahraoui et al., 2014) increased engraftment and efficacy of treatment. In mice undergoing pancreatic islet transplantation, treatment with IL-1RA gene therapy also led to better engraftment(Hsu et al., 2009) In humans, two trials including a total of 14 patients with type 1 diabetes mellitus, showed that treatment with anakinra during the first 1–2 weeks after the transplantation in combination with anti-thyomocyte globulin (ATG) and prednisone or etanercept/tacrolimus/MMF led to better engraftment and did not lead to increased risk of infections (Takita et al., 2012; Maffi et al., 2014).

In mice and rats undergoing high risk cornea transplantation, topical treatment with IL-1RA (Dana et al., 1997; Yamada et al., 1998; Dekaris et al., 1999; Jie et al., 2004a,b; Dana, 2007) or IL-1RA gene therapy (Yuan et al., 2012) reduced the incidence of graft dysfunction.

### The role of IL-1 in renal transplantation

In case of renal transplantation in patients with IL-1 related inflammatory disorders, inhibition of the increased IL-1 signaling is necessary to suppress the symptoms due to the inflammatory disease. It could be hypothesized that suppression of inflammation could also benefit the renal allograft, as the risk of possible impaired graft function or allograft rejection due to increased IL-1 serum levels may be important after transplantation. Studies on this subject show an association between increased IL-1 signaling and negative graft outcome.

In rats, during acute renal allograft rejection IL-1 mRNA is upregulated in kidney and spleen (Nagano et al., 1997) and IL-1 expression is upregulated in mononuclear, mesangial and endothelial cells in the graft (Tilney et al., 1993).

In humans, polymorphonuclear cells (PBMCs) (De Serres et al., 2011, 2012; Batal et al., 2014) and monocytes (Weimer et al., 2003) of patients with chronic rejection, glomerulonephritis or tubulopathy secrete more IL-1β than cells derived from non-rejecting patients or patients with normal renal graft biopsies. In patients with chronic renal graft dysfunction, the expression of IL-1β mRNA in the arterial wall of the renal arteries is upregulated (Zegarska et al., 2002).

Patients with acute renal allograft rejection have increased urinary excretion of IL-1β (Teppo et al., 1998). A single study showed higher serum IL-1β levels in patients with decreased graft function after transplantation (Caban et al., 2009) compared to patients with immediate graft function, although it has to be kept in mind that the concentration of circulating IL-1β and IL-1RA does not directly reflect the level of inflammation *in vivo*.

Polymorphisms in the genes encoding IL-1α, -β, and IL-1RA in renal transplant recipients are presumed to be associated with acute rejection (Manchanda et al., 2006; Manchanda and Mittal, 2008), short-term (Manchanda et al., 2006), and long-term graft function (Haldar et al., 1999), but this has not been reproduced in other studies (Marshall et al., 2000; Lee et al., 2004; Uboldi de Capei et al., 2004; Seyhun et al., 2012). This is most likely due to too small cohort sizes, differences in patient selection and case definition. Donor genotype does not influence graft outcome (Marshall et al., 2001).

Studies on the role of IL-1RA after renal transplantation show a protective effect. Low serum IL-1RA after transplantation is associated with delayed graft function (Sadeghi et al., 2003). Right before and during acute rejection, urinary excretion of IL-1RA was found to be decreased in some (Teppo et al., 1998, 2001; Xu et al., 2013), but not all studies (Sadeghi et al., 2003; Reinhold et al., 2012). These contradictory results may arise due to differences in patient selection, living or postmortal donors, immunosuppresive drug regimens, definition of allograft rejection, interval between transplant and rejection, or the cytokine measurement methods used. Higher urinary IL-1RA excretion is associated with better 1-year renal allograft function (Pereira et al., 2012) and the production of IL-1RA in renal biopsies taken before and during rejection is decreased compared to biopsies of non-rejecting kidneys (Oliveira et al., 1997; de Oliveira et al., 2002).

Renal transplantation is accompanied by ischemia-reperfusion injury, which may lead to delayed and impaired graft function. IL-1 is released during early reperfusion and induces apoptosis and an inflammatory response. There are few studies on the protective effects of IL-1RA in renal ischemia-reperfusion injury. During experimental ischemic clamping of murine native kidneys, one study showed that intraperitoneal treatment with a very high dose of IL-1RA (80 mg/kg) leads to significant reduction of tubular necrosis 24 h–5 days after ischemia-reperfusion and less apoptosis after 5 days (Rusai et al., 2008), but this has not been reproduced (Daemen et al., 2001), probably because of differences in the IL-1RA dose used and the methods used to detect ischemia-reperfusion injury. In a cardiac ischemia-reperfusion model in rabbits, intraventricular injection of 10–40 mg/kg IL-1RA after experimental myocardial ischemia decreased myocardial apoptosis (Li et al., 2004).

There are no studies on the effects of IL-RA treatment on reperfusion-ischemia injury in humans, and as the Il-1RA doses used in mice are high (approximately 4–300 mg/kg, compared to 2 mg/kg in children and a total of 100 mg in patients >40 kg in humans) these results cannot be directly extrapolated to a possible protective effect in humans. There are no reports on worse graft outcome after organ transplantation in patients with IL-1 driven inflammatory diseases, but as the number of patients with these kinds of diseases is limited, this might not be clinically evident. All three patients reported here had immediate graft function and no signs of rejection.

Besides possible worse graft outcome due to rejection or reperfusion-ischemia injury, the occurrence of secondary AA amyloidosis with consequent long-term graft loss due to prolonged uncontrolled inflammation in some IL-1 driven diseases is an additional reason why continuation of IL-1 inhibition after renal transplantation is important in patients with these diseases. Adequate inflammatory control protects against the development of AA amyloidosis.

## Conclusion

In our three patients that used IL-1 inhibitors before, during and after renal transplantation, continuing the IL-1 receptor antagonist anakinra on top of the standard immunosuppressive drug regimen with MMF, tacrolimus and prednisone was safe and well tolerated. Before and after transplantation the dose of anakinra should be adjusted to the renal function, as this influences clearance.

## Author contributions

CM wrote manuscript, performed literature search and review. MB provided case, corrected manuscript. FM provided case, corrected manuscript. AS provided case, corrected manuscript

### Conflict of interest statement

The authors declare that the research was conducted in the absence of any commercial or financial relationships that could be construed as a potential conflict of interest. The reviewer PS and handling Editor declared their shared affiliation, and the handling Editor states that the process nevertheless met the standards of a fair and objective review.

## Footnotes

^1^All three patients gave their consent for anonymous publication of their data.
